# Physician tracking in sub-Saharan Africa: current initiatives and opportunities

**DOI:** 10.1186/1478-4491-12-21

**Published:** 2014-04-23

**Authors:** Candice Chen, Sarah Baird, Katumba Ssentongo, Sinit Mehtsun, Emiola Oluwabunmi Olapade-Olaopa, Jim Scott, Nelson Sewankambo, Zohray Talib, Melissa Ward-Peterson, Damen Haile Mariam, Paschalis Rugarabamu

**Affiliations:** 1 The George Washington University, 2121 K Street NW, Suite 210, Washington, DC 20037, USA; 2 Uganda Medical and Dental Practitioners’ Council, Kampala, Uganda; 3 Ibadan, Nigeria and African Centre for Global Health and Social Transformation, University of Ibadan, Kampala, Uganda; 4 Makerere University College of Health Sciences, Kampala, Uganda; 5 Florida International University Herbert Wertheim College of Medicine, Miami, FL, USA; 6 Addis Ababa University, Addis Ababa, Ethiopia; 7 Dar es Salaam, Tanzania and African Centre for Global Health and Social Transformation, Hubert Kairuki Memorial University, Kampala, Uganda

**Keywords:** Health workforce, Human resources for health, Physician tracking systems, Sub-Saharan Africa, Medical education

## Abstract

**Background:**

Physician tracking systems are critical for health workforce planning as well as for activities to ensure quality health care - such as physician regulation, education, and emergency response. However, information on current systems for physician tracking in sub-Saharan Africa is limited. The objective of this study is to provide information on the current state of physician tracking systems in the region, highlighting emerging themes and innovative practices.

**Methods:**

This study included a review of the literature, an online search for physician licensing systems, and a document review of publicly available physician registration forms for sub-Saharan African countries. Primary data on physician tracking activities was collected as part of the Medical Education Partnership Initiative (MEPI) - through two rounds over two years of annual surveys to 13 medical schools in 12 sub-Saharan countries. Two innovations were identified during two MEPI school site visits in Uganda and Ghana.

**Results:**

Out of twelve countries, nine had existing frameworks for physician tracking through licensing requirements. Most countries collected basic demographic information: name, address, date of birth, nationality/citizenship, and training institution. Practice information was less frequently collected. The most frequently collected practice fields were specialty/degree and current title/position. Location of employment and name and sector of current employer were less frequently collected. Many medical schools are taking steps to implement graduate tracking systems. We also highlight two innovative practices: mobile technology access to physician registries in Uganda and MDNet, a public-private partnership providing free mobile-to-mobile voice and text messages to all doctors registered with the Ghana Medical Association.

**Conclusion:**

While physician tracking systems vary widely between countries and a number of challenges remain, there appears to be increasing interest in developing these systems and many innovative developments in the area. Opportunities exist to expand these systems in a more coordinated manner that will ultimately lead to better workforce planning, implementation of the workforce, and better health.

## Introduction

The World Health Organization estimates there is a global shortage of nearly 2.3 million physicians, nurses, and midwives; and in the most challenged countries in sub-Saharan Africa (SSA), workforce would need to be scaled up by almost 140% in order to meet international health targets such as the Millennium Development Goals [1]. More than half of countries in SSA have fewer than ten physicians per 100,000 people compared to the United States, Canada, and most of Europe that average more than 250 physicians per 100,000 [2].

The information above is well known to those working in global health and human resources for health. But the questions remain: How many actual doctors are practicing in SSA? How many medical school graduates are retained? How many more are needed? Research on current and needed physician workforce in SSA has begun to emerge in the last decade. Studies generally used physician licensing databases available from medical councils and ministries of health, training institution records, or population census data to project the supply and demand of physicians and to draw conclusions related to geographic maldistribution [3-8]. Some studies used regional databases to draw conclusions related to physician emigration from one region to another [9,10]. These studies also highlighted the challenges that remain in tracking physicians. Data on health workers entering, exiting and moving within and between systems is limited [11]. In many countries, the most established databases of physicians are Medical Council registration systems. However, the primary goal of Medical Councils has been to regulate the medical profession [12] and the data gathered for this purpose is not always matched to the needs of workforce planning. Therefore, human resources information systems are varied and often not fully used for workforce planning and decision-making [13]. Lack of standardized information is hindering the ability of governments and international organizations to intervene or understand the effects of their investments.

Having comprehensive, scalable and sharable systems for tracking detailed information about health care workers is critical for effective and efficient workforce planning. Knowing the numbers, specialties, and the geographic distribution of doctors is essential to increasing the absolute numbers, increasing access to health care, and correcting historic and geographic inequities. Tracking systems are important to evaluate the successes and failures of interventions to train and retain health care workers in high need specialties and recruit and inspire those who will serve in rural and underserved communities [14]. Tracking systems are also critical for emergency response - for public health reporting, emergency notification, and mobilization of health care workers in a humanitarian or environmental crisis. Finally, tracking systems can be used to promote quality health care, through wide-scale information sharing and continuing medical education [15,16].

Efforts are underway to support human resources for health tracking and evaluation. This study provides a detailed examination of current physician tracking systems, challenges involved, opportunities in SSA, and specifically the role of medical schools in contributing to developing tracking systems. This review is instructive both for developing physician tracking systems that can inform workforce and other health policies, as well as for developing tracking systems and policies around other critical health care workers.

## Methodology

We conducted an environmental scan for country level physician tracking systems in SSA. The environmental scan included a review of the literature, online search for physician licensing systems, and document review of publicly available physician registration forms for SSA countries. We used the Scopus database to search for articles fitting the following criteria: 1) detailing the use of human resource for health data for the purpose of physician tracking; 2) detailing implementation of health management information systems or human resources information systems for the purposes of developing physician and provider databases; or 3) describing the role of national institutions, such as medical councils and ministries of health, in physician tracking activities. Initial search results yielded 2,177 articles. After a review of the abstracts and elimination of duplicates, 165 articles were retained. Full text review yielded 63 relevant articles.

We searched Ministry of Health and Medical Council web sites for descriptions of registration systems, requirements, and registration forms. Data fields in each form were compared to determine commonalities and variations across the information required for physician registration across countries.

Primary data on physician tracking activities in SSA countries was collected as part of the Medical Education Partnership Initiative (MEPI). MEPI is a US government funded grant program to 13 SSA medical schools in 12 different countries to support capacity development of health professions training in the region. MEPI also supports a coordinating centre, which conducts an annual survey and annual site visits to all MEPI schools as part of its monitoring and evaluation role. This analysis utilizes data from the first two rounds of the MEPI annual survey and site visits.

The annual survey collects data from all 13 MEPI direct grantees, as well as other medical schools within their countries with whom they have formed consortia for their MEPI grant. The analysis in this paper focuses on the 13 MEPI direct grantees. Related to the issue of tracking, the MEPI annual survey asks detailed questions on graduate tracking of schools at baseline and asks for any changes in this information at round 2. In addition, the round 2 MEPI annual survey asked whether a national database of physicians existed. Round 1 of the survey, which serves as a baseline for the MEPI program, was implemented between 9 February 2011 and 31 March 2011, largely before implementation of MEPI activities began. Round 2 of the MEPI annual survey was implemented between 23 April 2012 and 15 June 2012. MEPI school principal investigators provided retrospective permission to use the survey data for this publication.

MEPI site visits took place around the same time, with round 1 visits from 10 January 2011 to 26 August, 2011 and round 2 visits from 6 February 2012 to 27 June 2012.

In this analysis we highlight two specific innovations in Ghana and Uganda that were identified through site visits. In each case, additional information was sought after site visits. In the case of Ghana, the data for the report was mainly obtained through a WHO report that was published highlighting the initiative. In the case of Uganda, one of the authors is with the Uganda Medical and Dental Practitioners’ Council and helped to found the messaging system described.

## Findings

### Environmental scan

Review of the literature and information available online from national institutions, provided details of existing physician tracking structures in a sample of SSA countries, focused on MEPI countries (Table 1) [17-31]. Of the twelve MEPI countries, nine had existing frameworks for physician tracking through licensing requirements, generally by medical councils funded by ministries of health. In all MEPI countries, medical councils operated independently of ministries of health, although many were linked through funding or personnel. All nine countries with existing tracking frameworks required physician registration, although enforcement mechanisms were often absent. For instance, according to the Medical and Dental Council of Nigeria, the total number of Nigerian doctors registered in December 2008 was about 52,000. However, only 12,344 professionals renewed their obligatory annual practicing licenses that year [31].

**Table 1:** **Physician registration requirements in 12 sub-Saharan African countries**^**1**^

***Characteristic***	***Number of countries***
Obligatory registration	9
Annual renewal	8
Electronic databases	3
Medical student registration	3

^1^Countries included: Botswana, Ethiopia, Ghana, Kenya, Malawi, Mozambique, Nigeria, South Africa, Tanzania, Uganda, Zambia, and Zimbabwe.

Registration forms were accessible online for eleven SSA countries: Botswana, Ghana, Kenya, Malawi, Namibia, Nigeria, South Africa, Tanzania, Uganda, Zambia, and Zimbabwe (Table 2) [32-42]. The majority of forms included basic demographic information: name, address, date of birth, nationality/citizenship, and training institution. Practice information was less frequently collected. The most frequently collected practice fields were specialty/degree and current title/position. However, location of employment and name of current employer were less frequently collected and few collected information on sector of employment. No country required registrants to include the average number of hours worked per week.

**Table 2:** **Physician registration data collection fields in 11 sub-Saharan African countries**^**1**^

***Field type***	***Number of countries from sample with field included on registration form***
Name	11
Address	11
Nationality/citizenship	10
Professional registration number	8
Specialty/degree	9
Training institution	9
Date of birth	9
Current title/position	7
Gender	7
Location of employment	6
Name of current employer	6
Marital status	5
Passport/ID number	3
Sector of employment (public/private)	2
English proficiency	2
Country of training	2
Race	2
Hours worked per week	0

^1^Countries included: Botswana, Ghana, Kenya, Malawi, Namibia, Nigeria, South Africa, Tanzania, Uganda, Zambia, and Zimbabwe.

Findings relevant to changing country health systems are of particular note. Only two countries collect information on public/private sector of employment, yet all 11 countries have documented private sectors. Additionally, all 11 countries are conducting post-graduate medical education training, yet two countries do not request information regarding the registrant’s specialty or degree.

### MEPI annual survey

Data from the MEPI annual survey showed that at baseline no reporting MEPI school had a system in place to track graduates. However, the majority of the schools reported plans to implement a system in the future. Only one school reported undertaking exit interviews, while six schools maintained some sort of information for graduates. The most common method used to maintain contact with graduates was alumni associations, followed by alumni reunions, alumni social events, and continuing medical education. No schools reported using social media or newsletters.

By round 2, the MEPI schools had made substantial progress. Since baseline, six schools reported taking steps to implement a system to track graduates. Four schools reported progress in constructing a database, one school was utilizing surveys to track past students, while another reported working with district officials to get more information on past medical school graduates. Schools continued to report using alumni networks to maintain contact with graduates, but social media appeared to be catching on with five schools reporting use of social media to maintain contact with graduates. Twelve of the schools also reported that their country has a national database of physicians, with five schools indicating they could access the database to locate graduates.

### Innovation: mobile technology access to physician registries in Uganda

The Uganda Medical and Dental Practitioners’ Council (UMDPC), is a statutory body mandated to register all medical and dental practitioners in the country, supervise training institutions and enforce standards of practice. Longitudinal tracking of the distribution, movement, and performance of physicians in the health system and training institutions remains a challenge but UMDPC has introduced innovative efforts to minimize these deficiencies using mobile phones.

UMDPC established computerized physician data and registers in 2007 to make it easier to track practitioners (inflows, outflows, and location), their qualifications and complaints received from the public. In 2011, the council introduced mobile phone short messaging system (SMS)-based reference directories of doctors, health facilities at all levels (including clinics and hospitals) and health training institutions. Any member of the public can text dedicated numbers using mobile phones and check which practitioners are registered to practice in particular areas and which clinics are licensed to operate in various localities. The system was developed through a public-private partnership. Development costs were supported by the SMS provider. Consumers are charged 200 Ugandan shillings (eight cents in US$) and the fee is split between the telephone company (100 Ugandan shillings), SMS provider (50 Ugandan Shillings) and UMDPC (50 Ugandan shillings).

Consumers are also increasingly engaged in reporting illegally practicing providers and voicing their discontent on the standards of service delivery in their environments. The consumer calls are facilitating UMDPC to track failure to register and unprofessional behaviors among practitioners, and take corrective action. It is the expectation of UMDPC that as the system becomes more widely known and used by the consumers, the standards of professionalism among doctors and the quality of health care will improve.

### Innovation: MDNet in Ghana

In 2008, Switchboard (a US-based non-profit) [43] in partnership with Vodafone, a local telephone company, and the Ghana Medical Association (GMA) launched the Mobile Doctors Network (MDNet). This innovative public-private partnership provides free mobile-to-mobile voice and text messages to all doctors registered with the GMA. Approximately 2,200 physicians, nearly all physicians in Ghana, participate in the programme. Once the programme was established, Switchboard further produced and distributed the first-ever directory of all physicians in Ghana allowing doctors to expand their support network for medical advice and referrals. The start-up costs for this venture were minimal with the telecom providing the SIM cards and the GMA absorbing the cost of distributing the SIM cards to their members.

The partnership appears to be a win-win for all stakeholders: Vodafone, while having only 18% of the total market, has 100% of the physician market and has generated US$15 million in revenue from paid calls to family and friends. The GMA is benefitting as they have developed a one-way bulk SMS program that allows them to inform doctors of emergency situations and meetings. Physicians, who were spending upwards of US$70 per month on calls to each other for medical advice, can now make free unlimited calls to seek advice and guidance, they have an expanded support network and through the bulk SMS, are instantly informed of public health issues [44]. While the programme originated in Ghana, it has expanded to Liberia (with all 181 doctors registered) and recently, in February of 2013 to Tanzania where 9,000 doctors will have access to a similar service.

## Discussion

Physician tracking systems vary widely between countries - in data collected, frequency of data collection, and authority of the collecting body. However, momentum appears to be building to expand tracking systems and use these databases to support basic workforce planning activities as well as to advance the quality of health care through innovative practices.

Our review of the literature and existing physician registration systems in SSA suggests there is much variability in the physician tracking systems of different countries. While some are collecting more practice data, many still largely focus on basic demographic information. Expanded data collection will be needed to maximize physician and other health workforce planning activities. Our review also suggests a need to match data with changing health systems. For example, as private sectors grow in the region, information on which sector physicians are working in and the relative movement between the two sectors will be important to ensure appropriate access for all citizens. This information will act as a guide for students when considering their career prospects, for medical schools when reviewing their curricula and policies, and for governments when making policies and deciding on distribution of resources.

Findings from the MEPI survey suggest medical schools are beginning to embark on developing graduate tracking and alumni systems and many are already able to access information from their medical councils. Medical schools will be important stakeholders and partners in the development of country level physician tracking systems. Coordination of data collection and registration between medical schools and medical councils could significantly advance tracking systems by capturing all medical students into the country level physician tracking system and gathering additional background information that can be studied to understand what factors are related to desired workforce outcomes. Robust tracking systems will allow medical schools to track the outcomes of investments in their institutions, and close involvement in tracking systems is ultimately in the best interest of medical schools as HRH policies affecting medical schools will result from the workforce analysis and planning developed from these systems.

Further movement on tracking systems is occurring at country and regional levels. The African Health Workforce Observatory is supporting regional and country level development of health workforce information systems to inform human resources for health policies and initiatives [45].

Innovative approaches appear to be arising to address the challenge of how to incentivize physician participation in registration systems. The Uganda system incentivizes physician registration by making registration status available to patients, so that patients can make informed decisions about the doctors they choose. The Ghana MDNet programme creates an everyone wins situation where physicians benefit by being able to connect with other physicians, receive health alerts, and receive information on meetings; and the government and people benefit by having better informed physicians.

The Ghana MDNet programme demonstrates that technology can facilitate tracking systems as well as the thoughtful use of the systems to improve health. The MDNet programme’s use of mobile technology to provide health alerts to nearly all physicians in the country at once represents an advanced emergency response capability that rivals systems worldwide. Additional technology-based resources exist that will advance physician tracking systems. For example, Capacity*Plus* offers open source software that can be downloaded and adapted to country specific parameters [46]. Availability of such software means countries will not need to create new systems, and offers the opportunity to develop coordinated systems between countries to follow physicians regionally and globally.

It should also be noted that the increasing interest in SSA by international funding agencies means there are often multiple - sometimes fragmented and duplicative - efforts on offer that local parties must consider. In addition, local context, infrastructure, and resources must always be taken into account in adopting and adapting any of the described approaches to other locations.

## Conclusion

While physician tracking systems vary widely between countries and a number of challenges remain, there appears to be increasing interest in developing these systems and many innovative developments in the area. Opportunities exist to expand these systems in a more coordinated manner that will ultimately lead to better workforce planning, implementation of the workforce, and better health.

## Abbreviations

MEPI: Medical Education Partnership Initiative; WHO: The World Health Organization; SSA: Sub-Saharan African; UMDPC: The Uganda Medical and Dental Practitioners’ Council; SMS: Short messaging system; GMA: Ghana Medical Association.

## Competing interests

The authors declare that they have no competing interests.

## Authors’ contribution

CC carried out the coordination of the paper and outlined the manuscript. SB, KS, SM, EOO, JS, NS, ZT all contributed equally to drafting the manuscript. DHW and PR provided editorial assistance. All authors read and approved the final manuscript
